# Effect of an inactivated coronavirus disease 2019 vaccine, CoronaVac, on blood coagulation and glucose: a randomized, controlled, open-label phase IV clinical trial

**DOI:** 10.3389/fimmu.2023.1122651

**Published:** 2023-05-31

**Authors:** Qing Xu, Xi Lu, Xiaodong Liu, Yanwei Zhao, Dapeng Sun, Qingfan Cao, Haidong Liu, Tuantuan Yang, Yufei Song, Jingjing Lv, Ping Xiong, Jing Li, Jianwen Sun, Meng Xie, Yongjun Gao, Li Zhang

**Affiliations:** ^1^Shandong Provincial Key Laboratory of Infectious Disease Control and Prevention, Shandong Center for Disease Control and Prevention, Jinan, Shandong, China; ^2^Medical Affairs Department, Sinovac Biotech Co., Ltd., Beijing, China; ^3^Medical Affairs Department, Sinovac Life Sciences Co., Ltd., Beijing, China; ^4^Immunization Program Department, Rushan City Center for Disease Control and Prevention, Rushan, Shandong, China; ^5^School of Public Health, Shandong University, Jinan, Shandong, China

**Keywords:** SARS-CoV-2, CoronaVac, COVID-19 vaccines, thrombosis with thrombocytopenia syndrome (TTS), blood coagulation, blood glucose

## Abstract

**Background:**

Billions of doses of coronavirus disease 2019 (COVID-19) vaccines have been administered and several cases of thrombocytopenia with thrombosis syndrome (TTS) have been reported after the administration of adenoviral vector vaccines. However, the effects of an inactivated COVID-19 vaccine, CoronaVac, on coagulation are not well understood.

**Methods:**

In this randomized, controlled, open-label phase IV clinical trial, 270 participants including 135 adults aged 18–59 years and 135 adults aged 60 years or older, were enrolled and randomized to the CoronaVac group or to the control group in a 2:1 ratio and received two doses of CoronaVac or one dose of the 23-valent pneumococcal polysaccharide vaccine and one dose of inactivated hepatitis A vaccine on days 0 and 28, respectively. Adverse events were collected for 28 days after each dose. Blood samples were taken on days 0, 4, 14, 28, 32, 42, and 56 after the first dose to evaluate neutralizing antibody titers and laboratory parameters of coagulation function and blood glucose.

**Results:**

Fourteen days after the second dose of CoronaVac, the seroconversion rates of neutralizing antibodies against the prototype strain and beta, gamma, and delta variants of concern (VOC) of Severe Acute Respiratory Syndrome Coronavirus 2 (SARS-CoV-2) reached peak values of 89.31%, 23.3%, 45.3%, and 53.5%, respectively. The incidence of adverse reactions was 43.6% and 52.2% in the CoronaVac group and in the control group, respectively. All were mild or moderate in severity. For the laboratory parameters, there was no difference in the means of any parameter between the two groups at any time point, except for the D-dimer on day 14. However, the D-dimer in the CoronaVac group decreased on day 14 compared to the value at baseline, while a higher D-dimer value, instead of a decreased D-dimer value, was a risk factor for TTS.

**Conclusion:**

CoronaVac showed a good safety profile and could induce a humoral response against the prototype and VOCs of SARS-CoV-2 in adults 18 years or older, with no abnormal effects on laboratory parameters of blood glucose and coagulation function.

## 1 Introduction

The coronavirus disease 2019 (COVID-19) pandemic has led to millions of confirmed cases of COVID-19 worldwide and billions of vaccines against SARS-CoV-2 have been administered (1). These vaccines play an important role in protecting against COVID-19 infection, intensive care unit (ICU) admissions, and deaths (2–4). However, since March 2021, cases of thrombocytopenia with thrombosis syndrome (TTS) (also called vaccine-induced immune thrombocytopenia, VITT) have been reported in Europe and the United States after vaccination with adenoviral vector-based vaccines (5–7). The European Medicines Agency (8, 9), the Food and Drug Administration (10, 11), and the World Health Organization (12) then evaluated the cases and concluded that there may be was a causal relationship between the cases and vaccines.

TTS is a rare symptom characterized by acute venous or arterial thrombosis, thrombocytopenia, and positive PF4-heparin ELISA. The peak time for symptoms onset is 6 to 14 days after vaccination and the symptoms can range from mild adverse effects to death (13). The pathogenesis of TTS is not yet clear. The clinical diagnosis of TTS involves a combination of clinical evaluation, laboratory testing, and imaging studies. Laboratory tests may include platelet count and coagulation tests to evaluate thrombosis factors, and imaging studies could identify blood clots in the veins or arteries (13).

Inactivated COVID-19 vaccines have been widely used for vaccination around the world, but there have been few studies on TTS-related safety after vaccination. Therefore, there is an urgent need to study whether inactivated COVID-19 vaccines could affect coagulation function. Here, we report the results of a phase IV clinical trial to evaluate the effects of an inactivated vaccine (CoronaVac) on coagulation function, immunogenicity, and other adverse reactions. Fasting glucose was also detected and evaluated.

## 2 Methods

### 2.1 Study design and participants

This single-site, randomized, controlled, open-label phase IV clinical trial was conducted by the Shandong Provincial Center for Disease Control and Prevention (CDC) in Rushan, Shandong province, China (NCT04953325). Participants 18 years or older were recruited and randomized to evaluate the immunogenicity and safety of an inactivated COVID-19 vaccine (CoronaVac). The trial was approved by the Shandong Provincial CDC Ethics Committee and was conducted in accordance with the principles of the Declaration of Helsinki and Good Clinical Practice. All participants provided their written informed consent prior to screening.

### 2.2 Vaccines

Inactivated SARS-CoV-2 vaccine (CoronaVac), 23-valent pneumococcal polysaccharide vaccine (PPV23) and inactivated hepatitis A vaccine used in this trial were manufactured by Sinovac Biotech Ltd. and approved by the China National Medical Products Administration (NMPA). CoronaVac is an inactivated COVID-19 vaccine. Each dose contains 600SU inactivated virus in 0.5 mL of aluminum hydroxide solution. The 23-valent pneumococcal polysaccharide vaccine contains 23 serotypes of pneumococcal capsular polysaccharide, each of which was 25 μg per dose (0.5 mL). The inactivated hepatitis A (HepA) vaccine contains 500 SU inactivated hepatitis A virus per dose in 1 mL of aluminum hydroxide solution.

### 2.3 Procedures

Participants aged 18 years or older were selected by an oral consultant according to inclusion and exclusion criteria. Criteria for critical exclusion included participants with 1) a history of SARS-CoV-2 infection or vaccination, 2) severe chronic diseases, 3) abnormal fasting blood glucose or diabetes, or 4) diagnosed coagulation abnormalities or diseases prone to thrombosis or bleeding. Other detailed inclusion and exclusion criteria are listed on ClinicalTrials.gov (NCT04953325).

A total of 270 participants were recruited and randomly assigned to the CoronaVac group and the control group in a 2:1 ratio. There were 180 participants in the CoronaVac group (including 90 adults aged 18–59 years and 90 adults aged 60 years or older) who received two doses of CoronaVac in a 28-day interval and 90 participants (including 45 adults aged 18–59 years and 45 adults aged 60 years or older) in the control group who received one dose of PPV23 on day 0 and one dose of inactivated HepA vaccine on day 28.

For safety assessment, participants were required to record the solicited adverse events on paper for the first 7 days after each dose and the unsolicited adverse events for 28 days after each dose. Serious adverse events were recorded throughout the trial. Adverse events were classified according to the guidelines released by the China NMPA (14, 15) and the US Department of Health and Human Services (HHS) (16).Blood samples were collected on days 0, 4, 14, 28, 32, 42, and 56 to evaluate the neutralizing antibody titers and laboratory parameters.

### 2.4 Outcomes

The primary safety outcome was abnormal differences in the means of laboratory parameters between the CoronaVac group and the control group at seven time points. Laboratory parameters included fasting blood glucose, platelet count, prothrombin time (PT), activated partial thromboplastin time (APTT), thrombin time (TT), fibrinogen (FIB), international normalized ratio (INR), D-dimer, erythrocyte sedimentation rate (ESR), and anti-heparin/platelet factor 4 antibody. Laboratory parameters adverse events were evaluated according to the China NMPA guidelines and the US HHS tables (14–16).

The second safety outcome was adverse reactions within 7 and 28 days after each dose. The primary immunogenic outcome was the seroconversion rate of the neutralizing antibody against the prototype strain of SARS-CoV-2 at 28 days after the second dose of CoronaVac. The secondary immunogenic outcomes included geometric mean titers (GMT), geometric mean increases (GMI), seroconversion rates (SCR), and seropositive rates of neutralizing antibodies against the prototype strain and VOCs of SARS-CoV-2 at different time points (days 0, 4, 14, 28, 32, 42, and 56). Seropositivity was defined as neutralizing antibody titer ≥ 8 and seronegative was defined as neutralizing antibody titer < 8. Seroconversion was defined as a change from seronegative at baseline to seropositive (17, 18).

### 2.5 Serum-neutralizing antibodies detection

The neutralizing antibodies against the prototype strain of SARS-CoV-2 (SARS-CoV-2/human/CHN/CN1/2020, GenBank number MT407649.1) and variants Beta (B.1.351), Gamma (P.1) and Delta (B.1.617.2) were quantified using a microcytopathogenic effect assay according to previous trials (17, 18). Serum samples were inactivated at 56°C for 30 minutes and serially diluted with cell culture medium. The diluted serum samples were incubated with equal volumes (50 µL) of live SARS-CoV-2 suspension for 2 hours at 37.0°C. Vero cells (1.0 × 10^5^ to 2.0 × 10^5^ cells per mL) were then added to the serum–virus suspensions in microplates and incubated at 36.5°C for 5 days. Cytopathic effects were recorded under microscopes and the neutralizing antibody titer was calculated by the dilution number of the 50% protective condition. These neutralizing antibody detections were performed by Sinovac Biotech Ltd. (Beijing, China).

### 2.6 Anti-platelet factor 4/Heparin antibody detection

The enzyme-linked immunosorbent assay (ELISA) kit for anti-heparin/platelet factor 4 antibodies (Anti-PF4) was purchased from Cloud-Clone Corp. (Wuhan, China). Serum samples were detected strictly according to the manufacturer’s instructions.

Standards or samples were added to the microtiter plate wells, which had been precoated with platelet factor 4/Heparin (PF4/H). Then after adding horseradish peroxidase (HRP) conjugated secondary antibodies, TMB substrate solution, and sulfuric acid solution step by step, the wells that contained anti-PF4/H antibodies would change colors to yellow. The concentrations of the anti-PF4/H antibodies were calculated by the optical density (OD) values, which were measured by a spectrophotometer at 450 nm.

### 2.7 Glucose detection

The glucose hexokinase reagent kit was purchased from Medicalsystem Biotechnology Co., Ltd. (Ningbo, China). All samples were detected by the hexokinase/glucose-6-phosphate dehydrogenase method according to the manufacturer’s instructions.

### 2.8 Statistical analysis

Because there were no previous studies relevant to the primary safety outcome of the three vaccines used in this trial, no statistical methods were used to calculate the sample size. Based on practical considerations and guidelines in China that the minimum sample size for each group in a phase I clinical trial should be 20–30 individuals, 90 participants per CoronaVac age subgroup and 45 participants per control age subgroup (2:1) were determined. Data were entered into EpiData software (version 3.1, EpiData Association). Analysis methods were described in previous trials (17, 18). The safety set included all participants who received at least one dose of vaccine and the immunogenicity set included all participants who had received the two doses of vaccines and collected seven blood samples according to the protocol without violating. The 95% confidence interval (CI) for categorical data was calculated using the Clopper–Pearson method. The Pearson χ2 test or Fisher’s exact test were used to analyze categorical data. Then, 95% CI for GMT was calculated based on the standard normal distribution of the logarithmic transformation antibody titer, and the student’s t test was used to compare the logarithmic transformation antibody titer. The hypothesis testing was two-sided, and we considered p values of less than 0.05 to be significant. SAS software (version 9.4; SAS Institute) was used to analyze all results.

## 3 Results

### 3.1 Participants

**Figure 1:**
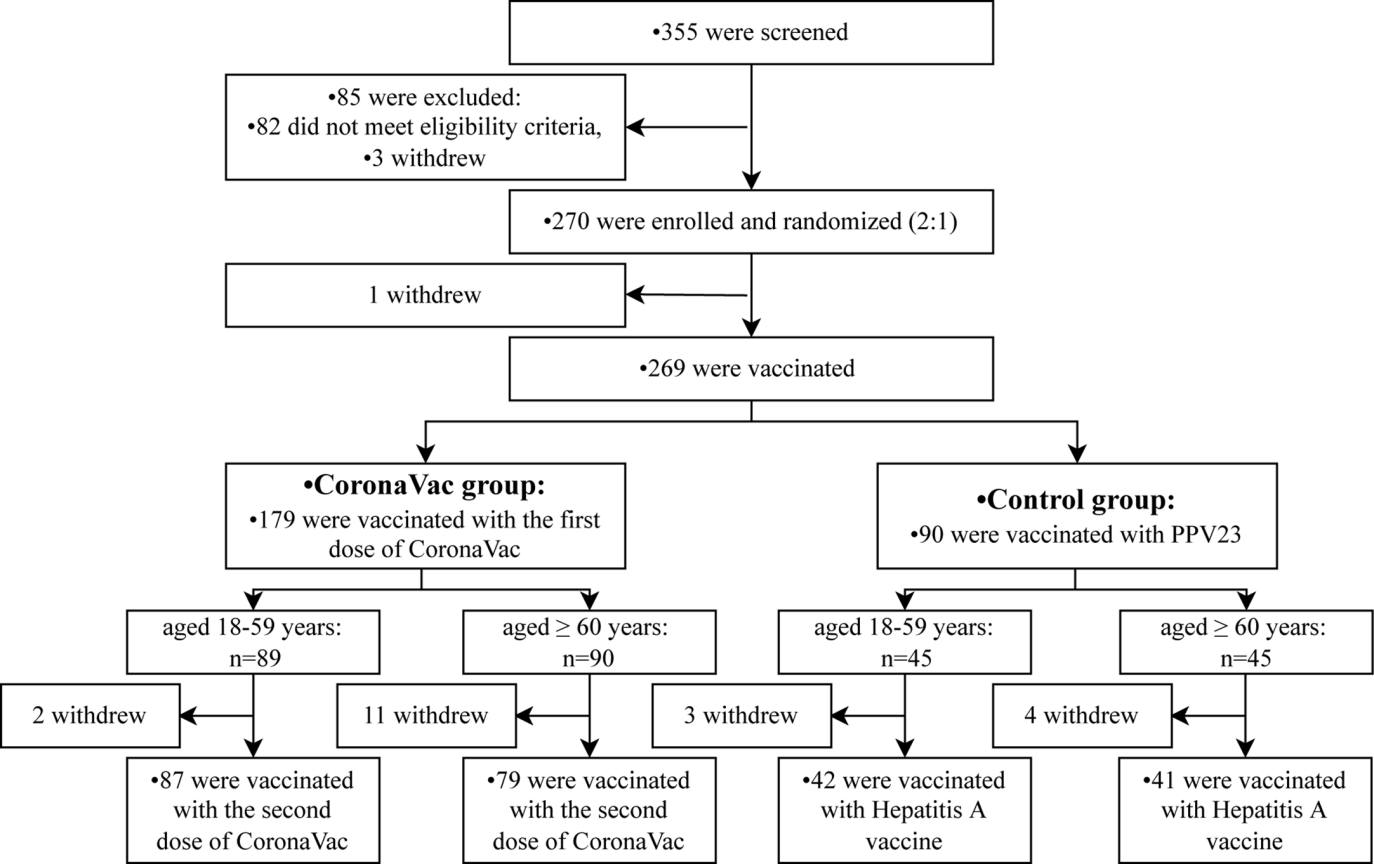
Study flow of the trial.

From 30 July to 25 August 2021, a total of 355 participants aged 18 years or older were selected, 270 participants were enrolled and underwent randomization. One hundred and eighty participants were assigned to the CoronaVac group (including 90 adults aged 18 to 59 years and 90 adults aged 60 years or older), and 90 participants were in the control group (including 45 adults aged 18 to 59 years and 45 adults aged 60 years or older). Of these participants, 269 were vaccinated with the first dose of CoronaVac or one dose of PPV23 on day 0, and 249 were vaccinated with the second dose of CoronaVac or a dose of the hepatitis A vaccine (**Figure 1**).

### 3.2 Demographic characteristics

At baseline, the demographic characteristics between the control group and the CoronaVac group were generally similar in terms of mean age, height, weight, sex, and ethnicity (**Table 1**). The mean age of the control group was 56.01 (SD 18.91) years; 65.56% were men and 34.44% were women. Most of the subjects were of Han ethnicity. The mean age of the CoronaVac group was 56.93 (SD 18.4) years; 57.22% were men and 42.78% were women. All subjects were of Han ethnicity.

**Table 1:** Demographic characteristics.

		Control group	CoronaVac group	P value
Total	No. of participants	90	179	
	Age, years	56.01 (18.91)	56.93 (18.4)	0.7012
	Height, cm	163.54 (10.91)	161.66 (9.78)	0.1525
	Weight, kg	67.36 (13.43)	67.7 (15.56)	0.8587
	Sex, Male	59 (65.56)	103 (57.22)	0.2051
	Sex, Female	31 (34.44)	76 (42.78)	
	Han ethnicity	89 (98.89)	179 (100)	0.3346
18-59 years	No. of participants	45	89	
	Age, years	40.2 (11.91)	41.43 (11.2)	0.5586
	Height, cm	168.84 (8.6)	167.3 (8.04)	0.308
	Weight, kg	70.73 (12.19)	74.21 (16.47)	0.1699
	Sex, Male	34 (75.56)	59 (66.29)	0.2718
	Sex, Female	11 (24.44)	30 (33.71)	
	Han ethnicity	44 (97.73)	89 (100)	0.3358
≥60 years	No. of participants	45	90	
	Age, years	71.82 (8.4)	72.27 (8.64)	0.7766
	Height, cm	158.24 (10.46)	156.08 (7.99)	0.2254
	Weight, kg	63.98 (13.88)	61.26 (11.48)	0.2287
	Sex, Male	25 (55.56)	44 (48.89)	0.4651
	Sex, Female	20 (44.44)	46 (51.11)	
	Han ethnicity	45 (100)	90 (100)	>0.9999

*Data are mean (SD) or n (%).

### 3.3 Immunogenicity

#### 3.3.1 GMTs and seroconversion rates of neutralizing antibodies against the prototype strain

**Figure 2:**
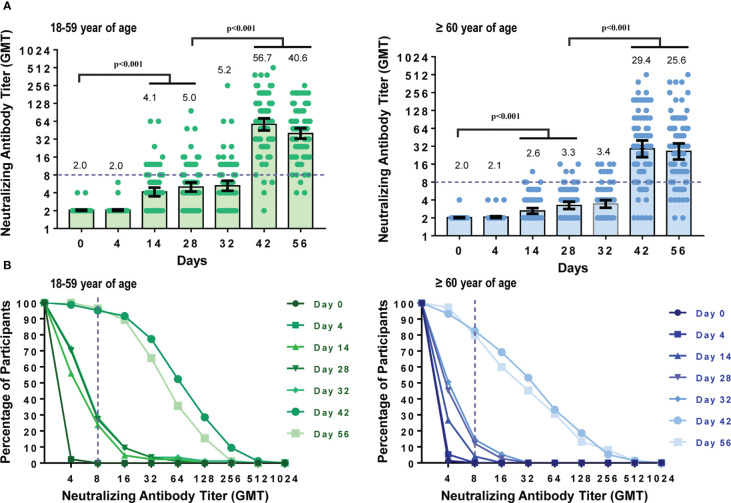
Dynamic changes in neutralizing antibody titers against prototype strain of SARS-CoV-2 at different time points. Participants received two doses of CoronaVac on Day 0 and Day 28. Neutralizing antibody titers at seven time points (Day 0, 4, 14, 28, 32, 42, 56) were measured. **(A)** GMTs of neutralizing antibodies against the prototype strain in participants aged 18–59 years or 60 years and older. Each dot represents an individual antibody titer, and each bar represents the GMT with 95%CI. **(B)** Reverse cumulative distribution curve for neutralizing antibody titers. The short-dashed line showed that the neutralizing antibody titer was 1:8, which means the participants seroconverted as all the participants were seronegative at baseline.

To assess the dynamic changes of neutralizing antibodies before and after immunization, blood samples from participants in the CoronaVac group were detected at seven time points. Four days after the first dose of CoronaVac, GMTs and seroconversion rates (SCR) of neutralizing antibodies against the prototype strain of SARS-CoV-2 showed no differences compared to those on day 0 in both the younger (18-59 years) (p_GMT_=0.705, p_SCR>_0.999) subgroups and older (60 years or older) (p_GMT_=0.083, p_SCR>_0.999) subgroups (**Figure 2A**). However,14 days and 28 days after the first dose, the neutralizing GMTs and seroconversion rates began to increase.

The neutralizing antibodies increased in a similar pattern after the second dose of CoronaVac. Neutralizing GMTs and seroconversion rates did not show significant differences between day 28 (before the second vaccination) and day 32 (4 days after the second dose) in each age subgroup (p_GMT-younger_=0.524, p_SCR-younger_=0.864, p_GMT-older_=0.342, p_SCR-older_=0.631) but increased significantly 14 and 28 days after the second dose (all the p-value were <0.001). Neutralizing antibody peaks were generated on day 42, with seroconversion rates reaching 89.31% (GMT 41.61 [95%CI 34.02–50.88], GMI 20.53 [95%CI 16.78–25.13]), 95.2% (GMT 56.7 [95%CI 44.82–71.82], GMI 27.90 [95%CI 22.0535.31]), and 82.7% (GMT 29.40 [95%CI 21.29–40.60], GMI 14.56 [95%CI 10.51–20.17]) in all participants, younger and older age subgroup, respectively. Because all participants were seronegative at baseline, the seropositive rate was the same as the seroconversion rate. When comparing GMTs and seroconversion rates of the two age subgroups, GMTs and seroconversion rates decreased with age on day 42 (p_GMT_=0.016, p_SCR_=0.010).

To further demonstrate the dynamic changes of the neutralizing antibody titers, reverse cumulative distribution plots were drawn (**Figure 2B**). As shown in **Figure 2B**, the neutralizing antibody titers increased significantly 14 and 28 days after each dose.

#### 3.3.2 GMTs and seroconversion rates of neutralizing antibodies against VOCs

To analyze the neutralizing antibody titers against beta, gamma, and delta variants of concern (VOC), blood samples from participants in the CoronaVac group were detected at four time points. All participants were seronegative and susceptible to any VOCs at baseline.

**Figure 3:**
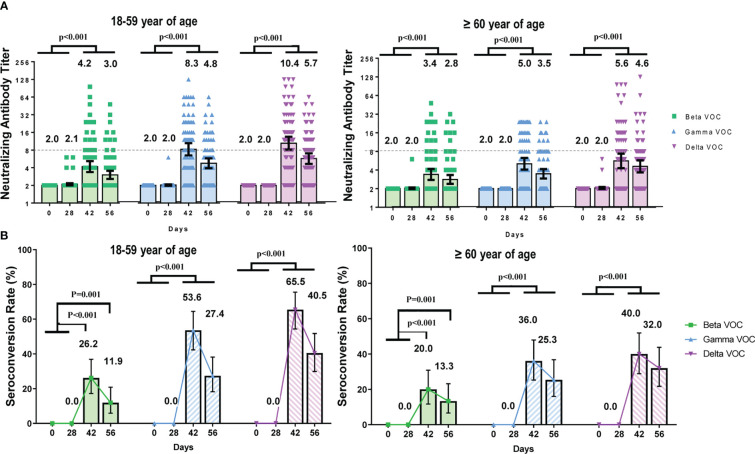
The dynamic changes of neutralizing antibody titers against SARS-CoV-2 VOCs at different time points. Participants received two doses of CoronaVac on Day 0 and Day 28 respectively and the neutralizing antibody titers were detected at four time points (Day 0, 28, 42, 56). **(A)** Geometric mean of neutralizing antibody titers in participants aged 18–59 and ≥ 60 years. Each dot represents an individual antibody titer, and the bar represents geometric mean titer with 95%CI. **(B)** Seroconversion rates of neutralizing antibody against VOCs at different time points. The lines showed the trends of the seroconversion rates.

The GMTs and seroconversion rates of neutralizing antibodies against SARS-CoV-2 VOC were lower than those against the prototype strain (**Figure 3**). GMTs and seroconversion rates did not show significant differences between baseline (day 0) and day 28(p_GMT_ ranged from 0.051 to >0.999) but increased significantly on day 42 and day 56(p_GMT_ were all <0.001, p_SCR_ were all ≤0.001). The GMTs of neutralizing antibodies against beta, gamma and delta VOCs on day 42 were 3.78 (95%CI 3.26–4.39), 6.54 (95%CI 5.54–7.73), 7.80 (95%CI 6.45–9.44) for all participants, with 11.0-, 6.4-, and 5.3-fold lower than the GMT against prototype strain, respectively. The seroconversion rates against beta, gamma and delta VOCs on day 42 were 23.27% (95%CI 16.9–30.6), 45.28% (95%CI 37.4–53.4), and 53.46% (95%CI 45.4–61.4) for all participants, with 3.8-fold, 2.0-fold, and 1.7-fold lower than the rate against the prototype strain respectively.

### 3.4 Safety

#### 3.4.1 Changes in laboratory parameters before and after vaccination

Ten parameters were detected at seven time points to assess the safety profile of CoronaVac in coagulation activity and blood glucose levels. The nine parameters related to coagulation function were platelet count, PT, APTT, TT, fibrinogen, INR, D-dimer, ESR and anti-heparin/platelet factor 4 antibody.

**Figure 4:**
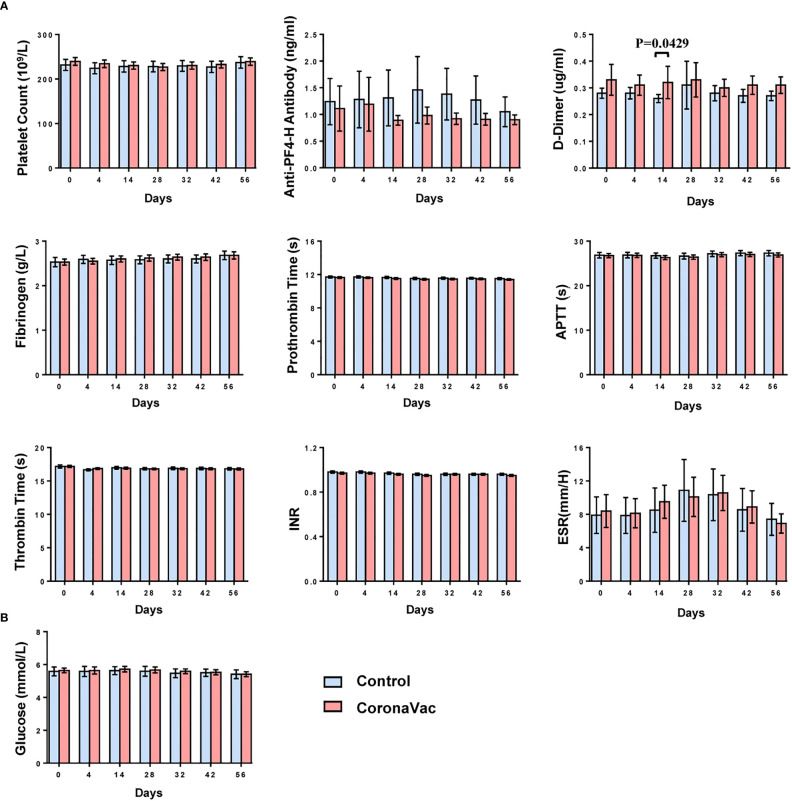
Dynamic changes in the means of laboratory parameters at each time point. **(A)** Nine parameters related to coagulation function. **(B)** Fasting blood glucose. The bar represents mean with 95%CI.

At each time point, the means of each laboratory parameter fell within the laboratory normal ranges (**Figure 4** and **Table S1**). Except for the D-dimer, there were no significant differences in the means of each parameter between the CoronaVac group and the control group. On day 14, the mean D-dimer in the CoronaVac group was higher than that in the control group (p=0.0429). However, comparing the mean D-dimer in the CoronaVac group on day 0 and day 14, the mean value decreased by 0.01 μg/mL (0.33 μg/mL on day 0 vs. 0.32 μg/mL on day 14). Considering that the decrease in D-dimer was not a risk factor for TTS, the difference was not of practical clinical significance. In terms of the D-dimer, at any other time point, there was no significant difference in the mean values between the two groups. No participant exhibited a markedly elevated D-dimer (>4 times the upper limit of normal), which is a criterion for diagnosing TTS (13).

To simulate the post-marketing vaccination condition, the oral consultation method was used, rather than laboratory tests, to screen participants. Therefore, subjects unaware of their chronic diseases, which were not contraindicated by vaccination, were recruited. After detecting laboratory parameters of blood samples, physicians evaluated whether laboratory results were laboratory abnormalities and clinically significant.

**Figure 5:**
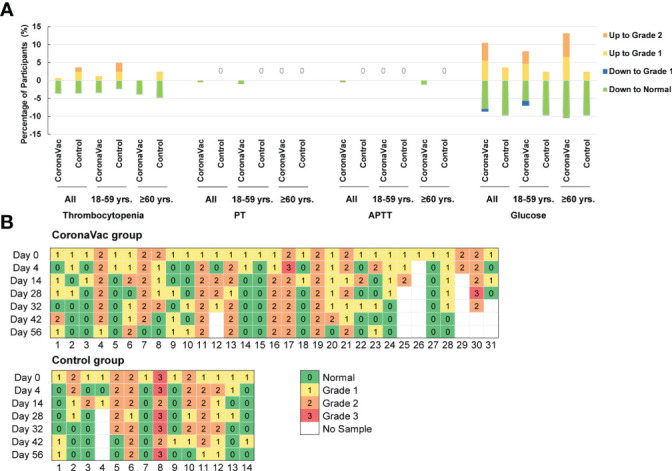
Grades Changes in the laboratory parameters between prevaccination and 28 days after the second dose in comparison with prevaccination. **(A)** Percentage of participants with upgraded or degraded laboratory abnormalities. ‘Up to Grade 1 or 2’ means the abnormality worsened to Grade 1 or 2 on day 56 compared to the severity at baseline; and ‘down to Grade 1 or normal’ means the abnormality relieved to grade 1 or normal on day 56 compared to the severity at baseline. **(B)** Grade changes in the blood glucose value of patients whose blood glucose values were abnormal at baseline. There were 31 participants were in the CoronaVac group, and 14 participants in the control group.

According to the relevant guidelines (14, 16), laboratory abnormalities of thrombocytopenia, PT, APTT, and fasting blood glucose were classified and analyzed. The proportions of participants with an upgraded abnormality were not significantly higher than those with a downgrade abnormality in each group when comparing the grade changes of each laboratory abnormality between prevaccination (day 0) and postvaccination (day 56) (**Figure 5A**) (p-value ranged from 0.121 to >0.999). Participants with abnormal blood glucose levels at baseline were focused, and no trend in vaccine-related risk with respect to altered glucose levels was found (**Figure 5B**). No severe laboratory abnormalities (≥ grade 3) were considered related to vaccines and no laboratory serious adverse events (SAE) were found in the trial.

#### 3.4.2 Adverse reactions

A total of 125 (46.5%) of the 269 participants reported at least one adverse reaction within 28 days of either vaccination, of which 78 participants were in the CoronaVac group and 47 participants were in the control group. In addition, 74 (55.2%) of the 134 participants aged 1859 years reported adverse reactions, which was higher than the percentage of participants aged 60 years or older (51 of 135, 37.8%) (p=0.004).

**Figure 6:**
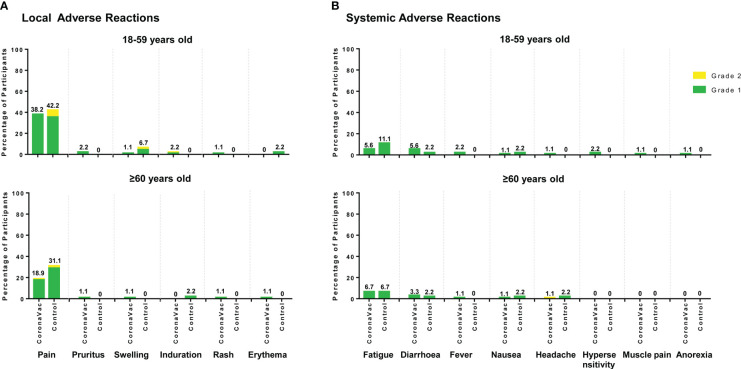
Local and systemic solicited adverse reactions reported for 28 days after either vaccination. **(A)** Local solicited adverse reactions; **(B)** Systemic solicited adverse reactions. The labels represent the total percentage of participants that reported the solicited adverse reactions of any grade.

All adverse reactions were grade 1 (42.8%) or grade 2 (3.7%) and the most common adverse reactions were injection site pain (28.5% in the CoronaVac group and 36.7% in the control group) and fatigue (6.1% in the CoronaVac group and 8.9% in the control group). There were no significant differences in the percentages of participants with any adverse reaction between the CoronaVac and the control group and between the two subgroups within the same age population. For the CoronaVac group, there were only three cases of grade 2, one case of induration at the vaccination site in participants aged 18–59 years, one case of pain at the vaccination site and one case of headache in participants aged 60 years or older (**Figure 6**). No grade 3 or higher adverse reactions were observed, and no serious adverse events were deemed related to any trial vaccine.

## 4 Discussion

In this phase IV clinical trial, two doses of CoronaVac with an interval of 28 days exhibited good immunogenicity and safety profiles. CoronaVac did not have any effect on laboratory parameters related to coagulation function and blood glucose.

Considering the vaccination policy in China stipulating that all participants who received post-marketing SARS-CoV-2 vaccines must know the lot number, this trial could not be blinded. For control vaccines, some studies have reported that Streptococcus pneumoniae infection could worsen the severity of COVID-19 (19, 20). Therefore, PPV23 was used to improve the benefits of the control participants. The HepA vaccine was used because it is also an inactivated aluminum adjuvant vaccine, the same as CoronaVac. These two types of vaccines have years of post-marketing safety data and no vaccine-related coagulation or blood glucose adverse reactions have been reported, so they are safe and suitable as control vaccines.

As a measure of immune system activation after vaccination, serum SARS-CoV-2 neutralizing antibodies were dynamically analyzed at seven time points after CoronaVac vaccination. Interestingly, we found that 4 days after either dose of CoronaVac, the neutralizing antibody level against the prototype strain did not show any difference compared to the prevaccination level of the corresponding dose. However, antibodies increased significantly 14 or 28 days after each dose. This indicated that it would take more than four days for each dose of CoronaVac to activate the humoral response. Our findings provide evidence supporting the statistical method used in previous studies evaluating the efficacy COVID-19 vaccines, whereby COVID-19 cases counted in the efficacy models occurred at least 14 days after the second dose of CoronaVac (3, 21).

CoronaVac vaccination could also activate neutralizing antibodies against the VOCs, however, it required a longer time. The GMT and seroconversion rate did not increase significantly until 42 days after the first dose (14 days after the second dose). Compared to the prototype strain, the values of GMT and the seroconversion rates against VOC decreased to different degrees. The greatest reduction was against beta VOC, followed by gamma and delta VOCs. Although antibody levels decreased, two doses of CoronaVac vaccines still showed effectiveness against different VOCs in real-world studies (22, 23). This indicated that the CoronaVac vaccine may induce a T-cell response and immunological memories of VOC that could then be activated after exposure to COVID-19, and they may also be activated after vaccination with a booster dose or a VOC vaccine.

According to the review published by the American Society of Hematology (ASH), five criteria were given to definitively diagnose TTS: 1) COVID-19 vaccination 4 to 42 days before symptom onset, 2) any venous or arterial thrombosis (often cerebral or abdominal), 3) thrombocytopenia, 4) positive for PF4 ‘HIT’ (heparin-induced thrombocytopenia) ELISA, 5) markedly elevated D-dimer (> 4 times the upper limit of normal) (13). Therefore, day 4 was chosen as the start to detect laboratory parameters after vaccination. Some studies also suggested that fibrinogen may decrease in cases of TTS (24–26). To systematically analyze the effects of CoronaVac on the coagulation function, nine laboratory parameters, which included all the parameters mentioned above, were finally chosen and detected at seven time points. To assess the effect on blood glucose, fasting blood glucose was detected simultaneously. The results showed that the changes in various laboratory parameters in the CoronaVac group were similar to those in the control group. No effect of the vaccine was found on TTS, coagulation, and blood glucose. In this trial, no vaccine-related risk factor for TTS was found.

Previous laboratory studies have also shown that CoronaVac did not cause thrombosis or TTS. Signorelli et al. (27) tested the lupus anticoagulant (LA), anticardiolipin (aCL), and anti-β2 glycoprotein I (aβ2GPI) antibodies in patients with primary antiphospholipid syndrome after vaccination and reported that CoronaVac did not cause thrombosis or induce changes in aPL production. Noikongdee et al. (28) conducted a cross-sectional study to evaluate anti-PF4/polyanionic antibodies in healthcare workers and found a low prevalence of anti-PF4 antibodies in Thais after CoronaVac vaccination. Liu et al. (29) showed that the inactivated COVID-19 vaccine had no effect on TTS by comparing the antibody status of aCL, aβ2GP1, anti-phosphatidylserine/prothrombin (aPS/PT), and anti-PF4-heparin before and 28 days after two-dose immunization. The results of these studies were similar to those of our study. And different from these studies, our study focused on the parameters involved in the clinical diagnosis of TTS according to the ASH standard. In addition, we evaluated multiple time points, not only a single time point after vaccination, so that we could study the parameter changes over time and systematically. Furthermore, these studies only reported laboratory indicators, we added the immunogenicity analysis of the vaccine at the same time as the context of the activation status of the immune system after vaccination, and 89.31% of the participants in this trial seroconverted.

Previous epidemiological findings in real-world studies did not report an increased risk of thrombocytopenia, thromboembolism, or diabetes associated with CoronaVac vaccinations (30–33). China’s national epidemiological data showed that the number of hospitalizations due to diabetes represented 2.2% of the total number of hospitalizations in 2020. In 2021, after the nationwide COVID-19 vaccinations, the proportion was also 2.2%. The data indicated that the COVID-19 vaccines did not cause diabetes (34). These results are also similar to those of our study. These epidemiological findings complemented the limitations of laboratory studies that laboratory studies could not allow large-scale observations due to limitations on blood sample collection and detection. And our study complemented the limitations of epidemiological results through in-depth analysis and validation of laboratory parameters.

Compared to the rigorous screening methods and criteria in typical phase I–III clinical studies, the enrolled population in this trial was closer to the actual vaccination population. Through laboratory test results before vaccination on day 0, we found that 17% of participants had an abnormal fasting glucose value higher than the normal range and some were suspected to be patients with diabetes. This showed that many vaccine recipients may not know their medical conditions well in the real world. Our findings provided the reference baseline data of the real-world population for the assessment of pharmacovigilance risk in China, indicating that some pharmacovigilance events diagnosed after vaccination may already had occurred before vaccination, and the percentage may not be as low as we thought before.

This trial has several limitations. Due to the lack of previous studies on laboratory parameters of the CoronaVac vaccine, the sample size of this trial was exploratory and small. As the incidence of TTS is very low, a TTS safety monitoring study in a larger population is required. Epidemiological studies of TTS are also available to further verify the effect of the CoronaVac vaccine on TTS in a larger population in China. Furthermore, in this study, neutralizing antibodies against Omicron VOC were not detected. They will be analyzed in the follow-up booster immunization study.

In conclusion, the CoronaVac vaccine could activate humoral responses against SARS-CoV-2 with a good safety profile and did not affect coagulation function and blood glucose levels.

## Data availability statement

The raw data supporting the conclusions of this article will be made available by the authors, without undue reservation.

## Ethics statement

The studies involving human participants were reviewed and approved by Shandong Provincial CDC Ethics Committee. The patients/participants provided their written informed consent to participate in this study.

## Author contributions

QX, XL, XDL, YZ, DS, YG, and LZ designed the clinical trial and the documents used in this trial. QC and HL organized the recruitment and visits of participants at the trial site. XL, XDL, TY, DS, JJL, PX, JS and MX monitored the progress of the trial. YS cleaned and analyzed the data. JL detected serum neutralizing antibodies and anti-PF4/H antibodies. QX, XL, and YZ drafted the manuscript. LZ and YG reviewed the manuscript. All authors contributed to the interpretation of the data and the critical review of the manuscript.
