# Supplementary material for: Effect of an inactivated coronavirus disease 2019 vaccine, CoronaVac, on blood coagulation and glucose: a randomized, controlled, open-label phase IV clinical trial
Source: Front Immunol. 2023 May 31;14:1122651. doi: 10.3389/fimmu.2023.1122651 (PMC10265469; doi:10.3389/fimmu.2023.1122651)
Supplement: Supplementary file 1 [file Table_1.docx]

Supplementary Material

# Supplementary Tables 1 Mean values of laboratory parameters at each time point

| Time | Group | Platelet | | Anti-PF4/H antibody | | D-dimer | | FIB | | PT | | APTT | | TT | | INR | | ESR | | Blood glucose | |
| --- | --- | --- | --- | --- | --- | --- | --- | --- | --- | --- | --- | --- | --- | --- | --- | --- | --- | --- | --- | --- | --- |
|  |  | Mean (10^9^/L) | p value | Mean (g/L) | p value | Mean (ug/ml) | p value | Mean (g/L) | p value | Mean (s) | p value | Mean (s) | p value | Mean (s) | p value | Mean | p value | Mean (mm/H) | p value | Mean (mmol/L) | p value |
| Day 0 | Control | 231.69 (58.34) | 0.299 | 1.24 (2.07) | 0.685 | 0.28 (0.09) | 0.1286 | 2.53 (0.5) | 0.9053 | 11.7 (0.58) | 0.4706 | 26.84 (2.99) | 0.8447 | 17.17 (1.11) | 0.9456 | 0.98 (0.05) | 0.4705 | 7.9 (10.41) | 0.7432 | 5.58 (1.30) | 0.6793 |
|  | CoronaVac | 239.67 (59.86) |  | 1.11 (2.86) |  | 0.33 (0.39) |  | 2.53 (0.47) |  | 11.64 (0.68) |  | 26.76 (3.11) |  | 17.18 (1.04) |  | 0.97 (0.06) |  | 8.39 (13.29) |  | 5.64 (0.97) |  |
| Day 4 | Control | 224.18 (57.22) | 0.1772 | 1.28 (2.45) | 0.7918 | 0.28 (0.1) | 0.1745 | 2.59 (0.42) | 0.422 | 11.71 (0.61) | 0.2979 | 26.86 (2.9) | 0.8574 | 16.67 (0.74) | 0.1173 | 0.98 (0.05) | 0.2719 | 7.86 (9.95) | 0.8547 | 5.58 (1.45) | 0.7341 |
|  | CoronaVac | 234.28 (55.85) |  | 1.19 (3.35) |  | 0.31 (0.25) |  | 2.55 (0.43) |  | 11.62 (0.65) |  | 26.79 (3.23) |  | 16.85 (0.88) |  | 0.97 (0.05) |  | 8.13 (11.58) |  | 5.64 (1.42) |  |
| Day 14 | Control | 228.29 (58.7) | 0.7957 | 1.31 (2.41) | 0.112 | 0.26 (0.07) | 0.0429 | 2.57 (0.43) | 0.5229 | 11.64 (0.6) | 0.1531 | 26.74 (2.71) | 0.2595 | 16.97 (0.91) | 0.6406 | 0.97 (0.05) | 0.1472 | 8.5 (12.24) | 0.5552 | 5.63 (1.10) | 0.5099 |
|  | CoronaVac | 230.20 (53.62) |  | 0.89 (0.6) |  | 0.32 (0.4) |  | 2.6 (0.46) |  | 11.52 (0.62) |  | 26.3 (2.98) |  | 16.91 (0.93) |  | 0.96 (0.05) |  | 9.51 (13.05) |  | 5.72 (1.10) |  |
| Day 28 | Control | 228.00 (53.97) | 0.8726 | 1.46 (2.86) | 0.139 | 0.31 (0.41) | 0.7611 | 2.58 (0.41) | 0.455 | 11.53 (0.58) | 0.2296 | 26.63 (2.96) | 0.5928 | 16.83 (0.82) | 0.7822 | 0.96 (0.05) | 0.2622 | 10.87 (16.96) | 0.7168 | 5.59 (1.38) | 0.628 |
|  | CoronaVac | 226.87 (51.72) |  | 0.98 (1.04) |  | 0.33 (0.42) |  | 2.62 (0.45) |  | 11.43 (0.61) |  | 26.41 (3.14) |  | 16.8 (0.85) |  | 0.95 (0.05) |  | 10.09 (15.37) |  | 5.67 (1.18) |  |
| Day 32 | Control | 229.43 (54.89) | 0.9064 | 1.38 (2.21) | 0.0671 | 0.28 (0.13) | 0.447 | 2.6 (0.41) | 0.4465 | 11.56 (0.57) | 0.2235 | 27.16 (2.82) | 0.6348 | 16.9 (0.87) | 0.5803 | 0.96 (0.05) | 0.217 | 10.34 (14.17) | 0.9063 | 5.47 (1.21) | 0.4316 |
|  | CoronaVac | 230.28 (53.15) |  | 0.92 (0.69) |  | 0.3 (0.21) |  | 2.64 (0.43) |  | 11.46 (0.65) |  | 26.97 (3.04) |  | 16.84 (0.81) |  | 0.96 (0.05) |  | 10.56 (13.74) |  | 5.59 (0.97) |  |
| Day 42 | Control | 227.18 (57.35) | 0.4498 | 1.27 (2.06) | 0.1235 | 0.27 (0.11) | 0.09 | 2.6 (0.4) | 0.5338 | 11.55 (0.5) | 0.2602 | 27.3 (2.63) | 0.4769 | 16.87 (0.86) | 0.714 | 0.96 (0.04) | 0.231 | 8.54 (11.68) | 0.8288 | 5.50 (1.01) | 0.803 |
|  | CoronaVac | 232.57 (49.72) |  | 0.91 (0.71) |  | 0.31 (0.22) |  | 2.64 (0.47) |  | 11.47 (0.61) |  | 27.02 (3.07) |  | 16.83 (0.84) |  | 0.96 (0.05) |  | 8.89 (12.43) |  | 5.53 (0.99) |  |
| Day 56 | Control | 237.21 (58.66) | 0.8002 | 1.05 (1.27) | 0.3127 | 0.27 (0.08) | 0.0745 | 2.68 (0.44) | 0.9279 | 11.5 (0.52) | 0.2211 | 27.31 (2.78) | 0.3053 | 16.82 (0.84) | 0.8091 | 0.96 (0.04) | 0.2424 | 7.4 (8.68) | 0.6471 | 5.41 (1.21) | 0.994 |
|  | CoronaVac | 239.10 (53.47) |  | 0.9 (0.58) |  | 0.31 (0.2) |  | 2.68 (0.52) |  | 11.4 (0.6) |  | 26.9 (3.02) |  | 16.79 (0.98) |  | 0.95 (0.05) |  | 6.91 (7.43) |  | 5.41 (0.93) |  |

Data are mean (SD).
